# 

**DOI:** 10.1192/bjb.2025.10114

**Published:** 2026-04

**Authors:** Kurt Wendelborn

**Affiliations:** Consultant Psychiatrist, Te Whatu Ora Health New Zealand Counties Manukau, Auckland, New Zealand.

**Figure:**
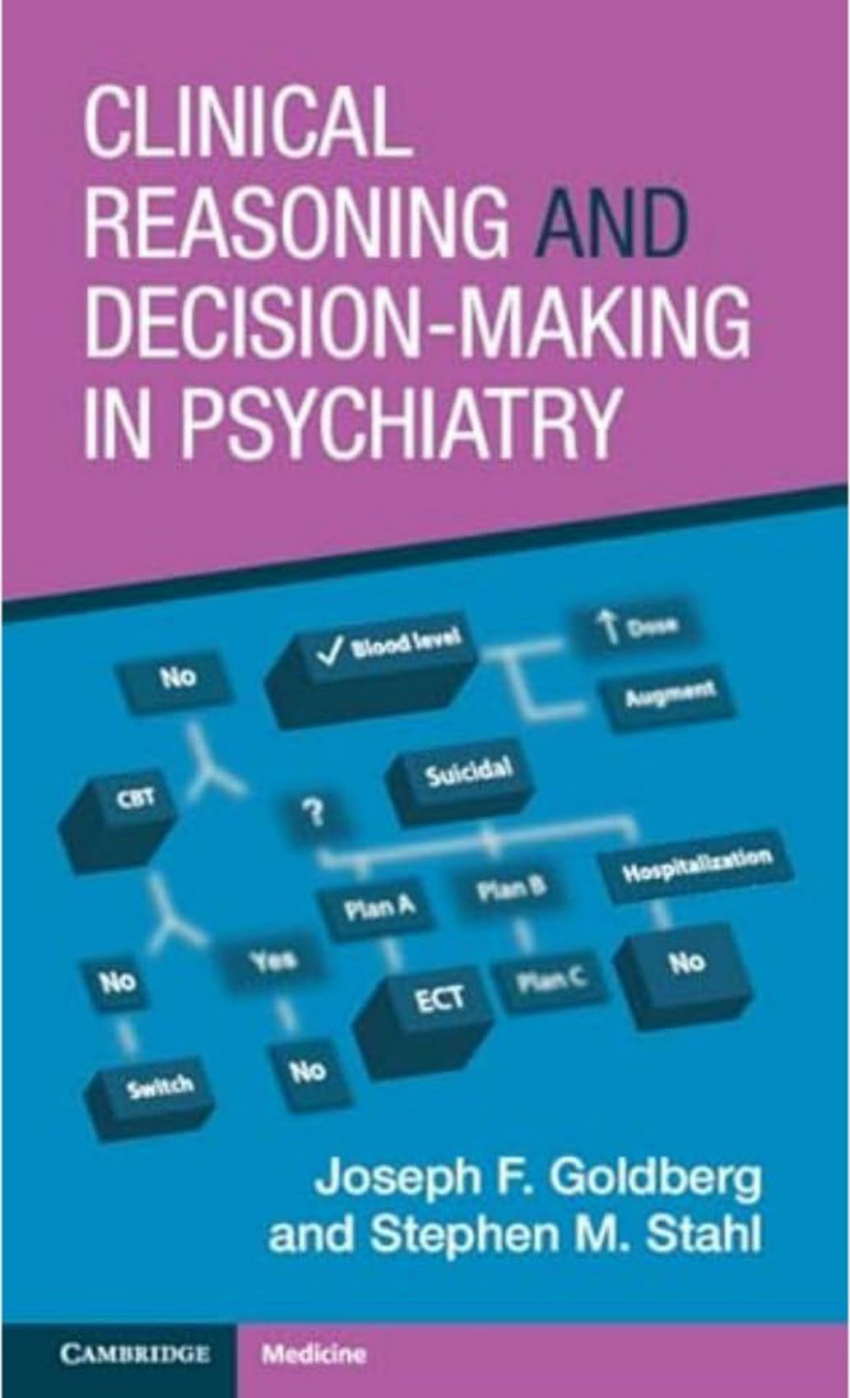

This book is a masterclass on how one might apply reasoned clinical decision-making amid the haste of workaday psychiatry. Throughout, the authors avoid advising clinicians on what they should do in any given scenario. Instead, their emphasis is on how clinicians and their patients might arrive at a route to travel by. Gathering and sifting information, establishing and maintaining the therapeutic alliance, identifying, weighing and planning any treatment stratagems, measuring any effects and deciding what to do next are all examined. There are excellent tables and diagrams, and comedically monikered psychiatrists apply themselves to a swathe of illustrative vignettes. There are also cute, often amusing, cartoons that sometimes illustrate and on other occasions add nuance to the main text.

Goldberg and Stahl are acknowledged doyennes in clinical psychopharmacology. The sections that focus on this are clearly and confidently presented, entirely up to date and extremely useful. I felt at times that more might have been said about potential adverse outcomes, for example for benzodiazepines when prescribed in circumstances without a clear therapeutic end-point. The authors touch on the debate about the place of long-term ‘stable-dose’ benzodiazepine prescribing but I would have appreciated more on this kind of perilous navigation. I was considerably heartened to read the recurring and salutary discussion of ‘therapeutic futility’. The closely related discussion about ‘deprescribing’ was also well framed.

The discussions of psychotherapy, however, unlike those of pharmacotherapy, draw on a more curious, occasionally anachronistic literature. For example, there is a discussion of paradoxical injunctions plucked from decades of relative obscurity that, although neatly illustrating the ambivalence that can afflict psychopathology and the therapeutic project, fails to acknowledge the ethical conundrum that this line of therapeutic intervention poses. This is of a piece with a discussion in the succeeding chapter on ‘deception and manipulation’, that at one point appears to make a case for ‘deliberate and beneficent deception’ in the service of recruiting the patient’s defences and excesses to a happier therapeutic outcome. A kindred niggling disquiet arose when reading the chapter on shared decision-making. Although well described, the subtext of this chapter read as if the authors felt that the ethos of shared decision-making was perhaps naive and doomed to failure or distortion.

One of the many pleasures of the book is the authors’ periodic admonishment to be wary of ‘apophenia’ in our prescribing or treatment planning, reminding us all that we can be as much a problem as a solution in our searching for patterns where none may exist. Is this then the book that might guide us through clinical reasoning and therapeutic decision-making? Perhaps not. Is it an excellent contribution towards that aspiration? Unequivocally.

